# Prolonged neuromuscular blockade by non-depolarizing neuromuscular blocking agents

**DOI:** 10.1186/s40981-019-0253-y

**Published:** 2019-05-19

**Authors:** Michiko Higashi, Takahiro Tamura, Yushi U. Adachi

**Affiliations:** 1 Department of Emergency and Medical Intensive Care Medicine, Nagoya University Hospital, 65 Tsurumai-cho, Showa-ku, Nagoya, 4668550 Japan; 2 Department of Surgical Intensive Care Medicine, Nagoya University Hospital, 65 Tsurumai-cho, Showa-ku, Nagoya, 4668550 Japan

To the Editor,

We read with great interest the case report by Moriwaki and Kayashima [1] in the journal. The authors described the patient demonstrating prolonged neuromuscular blockade even after administration of sugammadex considered as appropriate for reversal of rocuronium. After the surgery, the long-lasting residual blockade was antagonized by supplemental administration of neostigmine and atropine, and the patient’s trachea was extubated without any complications. Although the course and the explanation were acceptable, we have several concerns for the case.

First, the authors administered neostigmine and atropine. The reversal temporally increased the train of four ratio; however, no one can predict the duration of prolonging blockade and the half time of neostigmine is approximately 1 h. The patient showed the apparent residual blockade even after 163 min from the induction of anesthesia. More length of time might be required for the complete recovery [2]. Intensive care and careful observation would be appreciated.

Second, the authors emphasized the effect of magnesium and calcium-antagonists on acetylcholine release. The combination is well known for the treatment of cholinergic crisis in the area of toxicology [3]. The non-depolarizing neuromuscular blocking agents have presynaptic inhibitory effect on neuronal acetylcholine receptors [4]. The residual blockade might be so complicated and there was a room for discussion.

We previously reported the case of a patient developing recurarization after administration of sugammadex following a prolonged rocuronium infusion [5]. In the case, unexpected decrease of elimination of rocuronium by hypothermia might be a factor of the recurarization. The cause of unexpected prolongation and residual of neuromuscular blockade varies [6, 7]. Residual neuromuscular blockade absolutely could not be overlooked after anesthesia [8]. Anesthesiologists should pay more attention to prolonged neuromuscular blockade.
